# Micellar Liquid Chromatographic Determination of Carbaryl and 1-Naphthol in Water, Soil, and Vegetables

**DOI:** 10.1155/2012/809513

**Published:** 2012-02-09

**Authors:** Mei-Liang Chin-Chen, Maria Rambla-Alegre, Abhilasha Durgbanshi, Devasish Bose, Sandeep K. Mourya, Josep Esteve-Romero, Samuel Carda-Broch

**Affiliations:** ^1^Grup de Química Bioanalítica, Q.F.A., E.S.T.C.E., Universitat Jaume I, 12071 Castelló, Spain; ^2^Pfizer Analytical Research Centre, Ghent University, B-9000 Ghent, Belgium; ^3^Department of Applied Chemistry, IT, Banaras Hindu University, 221005 Varanasi, India; ^4^Department of Criminology and Forensic Sciences, Dr. H. S. Gour University, 470003 Sagar, India

## Abstract

A liquid chromatographic procedure has been developed for the determination of carbaryl, a phenyl-*N*-methylcarbamate, and its main metabolite 1-naphthol, using a C18 column (250 mm  ×  4.6 mm) with a micellar mobile phase and fluorescence detection at maximum excitation/emission wavelengths of 225/333 nm, respectively. In the optimization step, surfactants sodium dodecyl sulphate (SDS), Brij-35 and *N*-cetylpyridinium chloride monohydrate, and organic solvents propanol, butanol, and pentanol were considered. The selected mobile phase was 0.15 M SDS-6% (*v/v*)-pentanol-0.01 M NaH_2_PO_4_ buffered at pH 3. Validation studies, according to the ICH Tripartite Guideline, included linearity (*r* > 0.999), limit of detection (5 and 18 ng mL^−1^, for carbaryl and 1-naphthol, resp.), and limit of quantification (15 and 50 ng mL^−1^, for carbaryl and 1-naphthol, resp.), with intra- and interday precisions below 1%, and robustness parameters below 3%. The results show that the procedure was adequate for the routine analysis of these two compounds in water, soil, and vegetables samples.

## 1. Introduction

Carbaryl (1-naphthyl methylcarbamate) is one of the most widely and frequently used synthetic insecticides worldwide to eliminate chewing and sucking insects in a wide range of agricultural and domestic situations, including stored grain, ornamental plants, lawns, fruit, and vegetables, and around public buildings [1]. It is also used to control earthworms in turf, a growth regulator for the fruit thinning of apples and an animal ectoparasiticide. It is stable under neutral and weakly acidic conditions, and also to heat. In alkaline media, it hydrolyses to its main metabolite: 1-naphthol. Carbaryl can produce adverse effects in humans by skin contact, inhalation, or ingestion. In plants, only a small amount of carbaryl, which is deposited on outer layers, passes to tissues and is metabolized by hydrolysis or oxidation to several hydroxylated metabolites. Carbaryl applied to plants reaches soil and water [2], where it is metabolized by microorganisms or undergoes photodecomposition, with a half-life from 7 to 28 days depending on the aerobic/anaerobic character of the soil. Therefore, the simultaneous analysis of both carbaryl and 1-naphthol in water, soil, and vegetable samples is of interest.

 Methods for the analysis of carbaryl and 1-naphthol that are based on liquid chromatography [3–8] coupled with tandem mass spectrometry [6], fluorescence [4, 8], mass spectrometry [5, 7], a diode array detector [6, 7], gas chromatography [9, 10], micellar electrokinetic capillary electrophoresis [11, 12], capillary electrochromatography [13], spectrophotometry [14–16], and immunoassays [17, 18] have been reported. These techniques require the prior pretreatment by either liquid-liquid or solid phase extraction, for which chlorinated solvents are often used.

 Micellar liquid chromatography (MLC) includes mobile phases containing sodium dodecyl sulphate (SDS) as a surfactant at a concentration above its critical micellar concentration, and usually an organic solvent (propanol, butanol, or pentanol). This technique has proved useful in the determination of diverse groups of drugs [19].

 The present study is devoted to develop a useful analysis method for the widely used insecticide, carbaryl, and its major metabolite, 1-naphthol, in water, soil, and vegetables (lettuce) by MLC. Fluorescence detection enables great selectivity with the proposed method. In addition, this method is cost-effective and user-friendly since samples do not involve a pretreatment step. Finally, the developed procedure is useful for screening the studied compounds for agricultural, industrial, gastronomy, or environmental studies.

## 2. Experimental

### 2.1. Chemicals and Reagents

Carbaryl was supplied by Union Carbide (South Charleston, USA) and 1-naphthol was purchased from Sigma-Aldrich (Gillingham-Dorset, St. Louis, MO, USA). Stock standard solutions containing 100 *μ*g mL^−1^ of carbaryl and 1-naphthol were prepared. Pesticides were dissolved in few millilitres of methanol in an ultrasonic bath and were made up to the mark in the volumetric flask with SDS 0.15 M-6% (*v/v*) pentanol buffered at pH 3. Distilled-deionized water (Barnstead, Sybron, Boston, MA, USA) was used throughout, and solutions were stored at 4°C. Finally, they were conveniently diluted before the analysis.

 Micellar mobile phases were prepared by using sodium dodecyl sulphate of 99% purity purchased from Merck (Darmstadt, Germany), Brij-35 (Merck), or *N*-cetylpyridinium chloride monohydrate (NCPC) obtained from Across Organics (Geel, Belgium). The buffer salt was sodium dihydrogen phosphate (Merck), and 1-propanol, 1-butanol, or 1-pentanol, all of which were of HPLC grade, came from Scharlab (Barcelona, Spain) and were used as organic modifiers. All the solutions were filtered through 0.45 *μ*m nylon membranes (Micron Separations, Westboro, MA, USA) and stored at 4°C.

### 2.2. Apparatus

The chromatographic system consisted in an Agilent 1100 series high-performance liquid chromatograph (Agilent Technologies, Palo Alto, USA) equipped with a quaternary pump, an online degasser, an autosampler, a thermostated column compartment, and a fluorescence detector. Chromatographic signals were acquired and processed with the Agilent ChemStation software package (Revision B.03.01), which was used to obtain the chromatographic peak parameters (dead time, retention time, efficiencies, and asymmetry factors). These data were later processed with SPSS 17.0 (SPSS Inc., Chicago, IL, USA).

 All the pH measurements were made with a GLP 22 Crison pH-meter (Barcelona), provided with a combined Ag/AgCl/glass electrode. The analytical balance used was a Mettler-Toledo AX105 Delta-Range (Greifensee, Switzerland). The vortex shaker and sonification units were made by Selecta (Barcelona).

### 2.3. Chromatographic Conditions

Chromatographic separation of the pesticides was performed in a Kromasil C18 column (250 mm × 4.6 mm i.d., 5 *μ*m particle size) obtained from Scharlab. The selected mobile phase was 0.15 M SDS-6% (*v/v*) pentanol-0.01 M NaH_2_PO_4_ buffered at pH 3. The flow rate was 1 mL min^−1^, the injection volume was 20 *μ*L, and the column temperature was maintained at 25°C. The excitation and emission wavelengths selected for the detection of the studied compounds were 225 nm and 333 nm, respectively.

### 2.4. Sample Preparation

Carbaryl and 1-naphthol were added in water, soil, and vegetables. Water was directly added with carbaryl and 1-naphthol at the desired concentrations. Then 1 g of the soil or vegetable samples was weighed, finely ground using a mincer (Model MZ10, Petra Electric, Burgau, Germany), centrifuged at 5000 rpm for 5 min, mixed with 10 mL of 0.15 M SDS-6% (*v/v*) pentanol-pH 3, spiked with the pesticides, stirred for 10 min, and finally filtered directly into the autosampler vials through nylon membranes.

## 3. Results and Discussion

### 3.1. Fluorescence Detection

In the proposed mobile phase containing 0.15 M SDS-6% pentanol (*v/v*)-pH 3, detection in the ultraviolet region can be used only for the determination of carbaryl and 1-naphthol in water samples, where endogenous substances are not present and do not interfere. In the soil and vegetable matrices, however, some peaks were found and could interfere with the determination of both pesticides.

 The fluorescence detector is specific for carbaryl and 1-naphthol. The fluorescence signal reduces the broad band at the beginning of the chromatogram due to the endogenous compounds when these samples are injected. The chromatograms for the vegetable samples are similar to those obtained in water but do not show any endogenous compound. Compared to UV detection, fluorescence allows higher sensitivity (an increase in a factor of 6 in the micellar media) and selectivity. Based on the range of our study, carbaryl and 1-naphthol showed maximum excitation at 225 nm and emission at 333 nm. At these wavelengths, the endogenous substances present in soil and vegetables do not interfere with the determination of the pesticides studied.

### 3.2. Selection of the Mobile Phase

One important parameter for any analytical method is the selection of an optimum pH for the analysis. It is known that *N*-substituted derivatives of simple esters of carbamic acid are unstable, especially under alkaline conditions [20]. To avoid hydrolysis, we decided to carry out the analysis by buffering all the mobile phases at pH 3.

 Three surfactants were studied (anionic SDS, neutral Brij-35, and cationic NCPC) to select the most efficient one for pesticides separation. The main difference between them lies in the presence of negatively charged micelles in the SDS micellar mobile phases, micelles without a charge for the Brij-35, and those positively charged for NCPC [21].

 The usual behavior in MLC with pure micellar eluents (without an organic solvent) involves retention lessening when the surfactant concentration increases, which was observed for carbaryl and 1-naphthol in the mobile phases at pH 3 containing SDS, Brij-35 or NCPC. Surfactants NCPC and Brij-35 (Table 1) were discounted because retention was high and the efficiencies for both pesticides were poorer where compared to those obtained with SDS.

The retention of carbaryl and 1-naphthol in a C18 column with pure micellar eluents was high according to their octanol-water partition coefficient values (log *P*) of 2.36 and 2.85 (The Specialized Information Services) (Table 1). Thus, the addition of a small amount of an organic solvent helped shorten the retention times. The addition of propanol or butanol led to retention times being higher than 25 min. Therefore, pentanol was used as a modifier on account of its high elution strength because it was able to cut retention times to 15 min or less and offered the best efficiencies and asymmetry factors.

The optimization criterion was to obtain a mobile phase that allows the complete separation (maximum resolution) in an appropriate analysis time. Interpretative optimization strategies can be assisted by computer simulation with the *Michrom* software [22], which can graphically mimic the methodology followed by experienced chromatographers with less time and effort. The experimental design consisting in four mobile phases (located at the corners of a rectangular factor space) and buffered at pH 3 was used to examine the chromatographic behavior of the two pesticides. Thus, pesticides were injected into the following mobile phases, SDS (*M*)/pentanol (%, *v/v*): 0.05/2, 0.05/6, 0.15/2, and 0.15/6. Furthermore, the model employed for these predictions was [21]

(1) $$k=\frac{K_{AS}\left(1/\left(1+K_{AD}\varphi \right)\right)\;}{1+K_{AM}\left(\left(1+K_{MD}\varphi \right)/\left(1+K_{AD}\varphi \right)\right)\left[M\right]},$$

where [*M*] and **φ**are the concentrations of the surfactant and organic solvent, respectively; *K*_*AS*_ and *K*_*AM*_ correspond to the equilibria between the solute in bulk water and the stationary phase or micelle, respectively; *K*_*AD*_ and *K*_*MD*_ measure the relative variation in the solute concentration in bulk water and micelles given the presence of a modifier, as compared to a pure micellar solution (without a modifier). 

**Figure 1:**
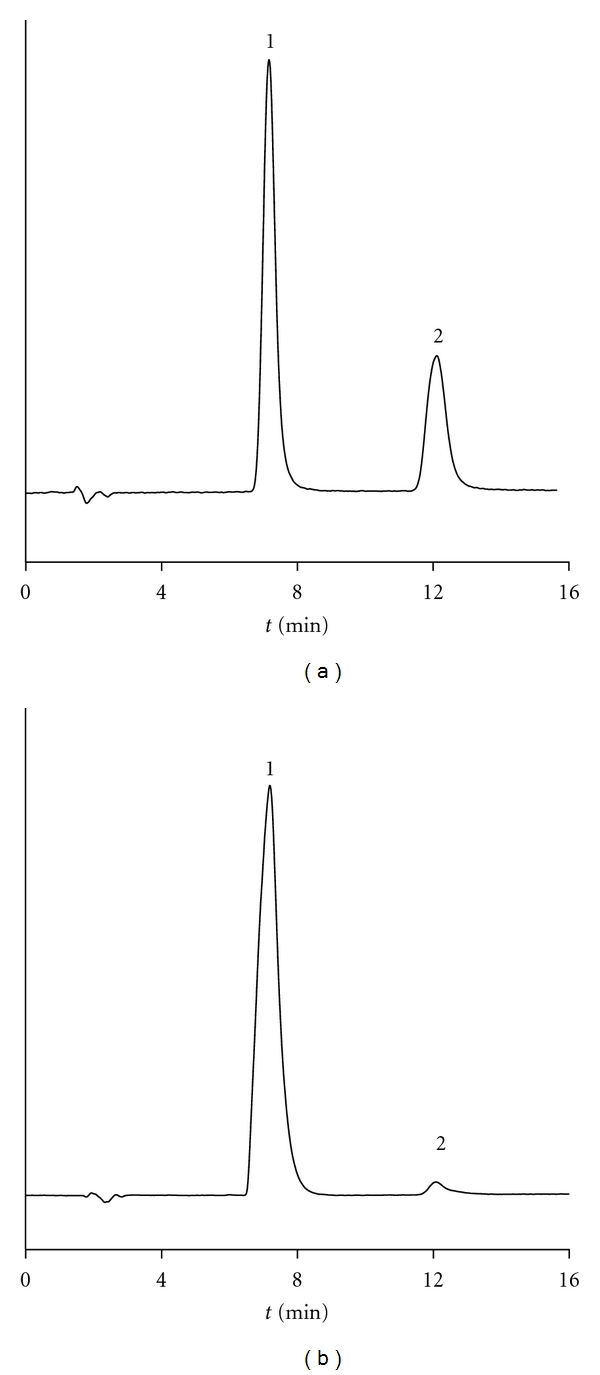
Chromatograms showing the separation of carbaryl (1) and 1-naphthol (2) using the mobile phase 0.15 M SDS-6% (*v/v*) pentanol- 0.01 M NaH_2_PO_4_ (pH 3). The injected samples were (a) water (after 10 days being spiked with 500 ng mL^−1^) and (b) soil (2 days after being spiked with 500 ng mL^−1^).

The best separation conditions were obtained by using a mobile phase composed of 0.15 M SDS-6% pentanol (*v/v*)-pH 3. Therefore, this mobile phase was selected as it was considered optimum; the chromatographic parameters (retention factor, retention time, efficiency, and asymmetry factor) for carbaryl and 1-naphthol were 4, 7.5, 4500, and 1.2, and 7.7, 12.5, 4500, and 1.2, respectively. Figures 1(a) and 1(b) show the chromatograms for the separation of carbaryl and 1-naphthol in water and soil, respectively.

### 3.3. Method Validation

The ICH-harmonized tripartite guideline [23] was followed to validate the method. The parameters studied were selectivity, linearity, limits of detection and quantification, accuracy, intra- and interday precisions, and robustness.

#### 3.3.1. Selectivity

To verify the absence of interfering endogenous compounds around the retention time of the analytes, 10 blank samples of the three matrices were analysed. No interference by the endogenous compounds was noted in the matrices studied.

#### 3.3.2. Linearity and Sensitivity

Using the selected chromatographic and detection conditions, the linear range of the signal response was studied over a concentration range of 15–1000 ng mL^−1^ for carbaryl and 50–1000 ng mL^−1^ for 1-naphthol. Standard solutions at eight different concentrations, 15 or 50, 100, 200, 400, 500, 600, 750, and 1000 ng mL^−1^, were prepared, conveniently diluted with 0.15 M SDS-6% (*v/v*) pentanol-pH 3 and added in the different matrices: water, soil, and vegetables. Each solution was injected into the chromatograph three times, and the average value of the peak areas was plotted against the concentrations. Data were adjusted for linear regression by the least mean squares method. All the calibration plots in the studied concentration range were linear with adequate correlation coefficients (*r* > 0.9999). The comparison made of the slopes and intercepts obtained in the three matrices, using Student's *t*-tests, demonstrates that there are no differences for the calibrations of carbaryl and 1-naphthol in the different samples. 

The limit of detection (LOD) was determined with the *3s criterion*, using the signal of ten blank samples injected separately. The limit of quantification (LOQ) was the lowest concentration used in the calibration curve. The slope, intercept, regression coefficient, LOD (ng mL^−1^), and LOQ (ng mL^−1^) values for carbaryl were 1248 ± 10, 0.12 ± 0.03, 0.99995, 5, and 15, and for 1-naphthol were 383 ± 3, 0.15 ± 0.03, 0.99998, 18, and 50, respectively. Calibration parameters and LODs and LOQs allowed the detection and quantification of carbaryl and 1-naphthol in the desired matrix without sample pretreatment.

#### 3.3.3. Precision and Accuracy

The intra- and interday precisions of the proposed method were studied at four known concentrations: 100, 200, 600, and 1000 ng mL^−1^ for carbaryl and 1-naphthol, added in water, soil, and vegetables. Intra- and interday precisions were evaluated by performing 20 consecutive injections either on the same day or over ten days over a 3-month period, respectively. The results, expressed as the percentage of the relative standard deviation (RSD, %), were below 2.5 and 3.2% for the intra- and interday precisions, respectively. The relative error (*E_r_*, %) of the measures was below 4%. These results are adequate for the routine analysis of both pesticides in water, soil, and vegetables.

#### 3.3.4. Robustness

The robustness of the method was examined by replicate injections (*n* = 6) of a standard solution of the pesticides at 100 ng mL^−1^ with slight changes made to the chromatographic parameters (surfactant concentration, percentage of pentanol, pH, and flow rate). Insignificant differences in the peak areas and less variability in the retention time or peak resolution were observed. The selected factors were not affected by the slight variations made to these parameters (with RSD (%) < 4.6), and the method using the recommended mobile phase (0.15 M SDS-6% pentanol (*v/v*)-pH 3) was robust. As expected, flow rate variation, and not the other parameters, had the greatest influence on the retention of the studied compounds.

### 3.4. Spiked Samples Analysis

Carbaryl and 1-naphthol, spiked into water at a concentration of 100 ng mL^−1^, were analysed. The two pesticides were also added to soil and vegetables (lettuce) taken from a field at a concentration of 100 ng g^−1^. After mixing, they were kept in a transparent non reactive container until analysed. The data obtained showed satisfactory recoveries for both compounds, and the results were in the 98.9–101.3% range.

### 3.5. Photodecomposition of Carbaryl in Water

**Figure 2:**
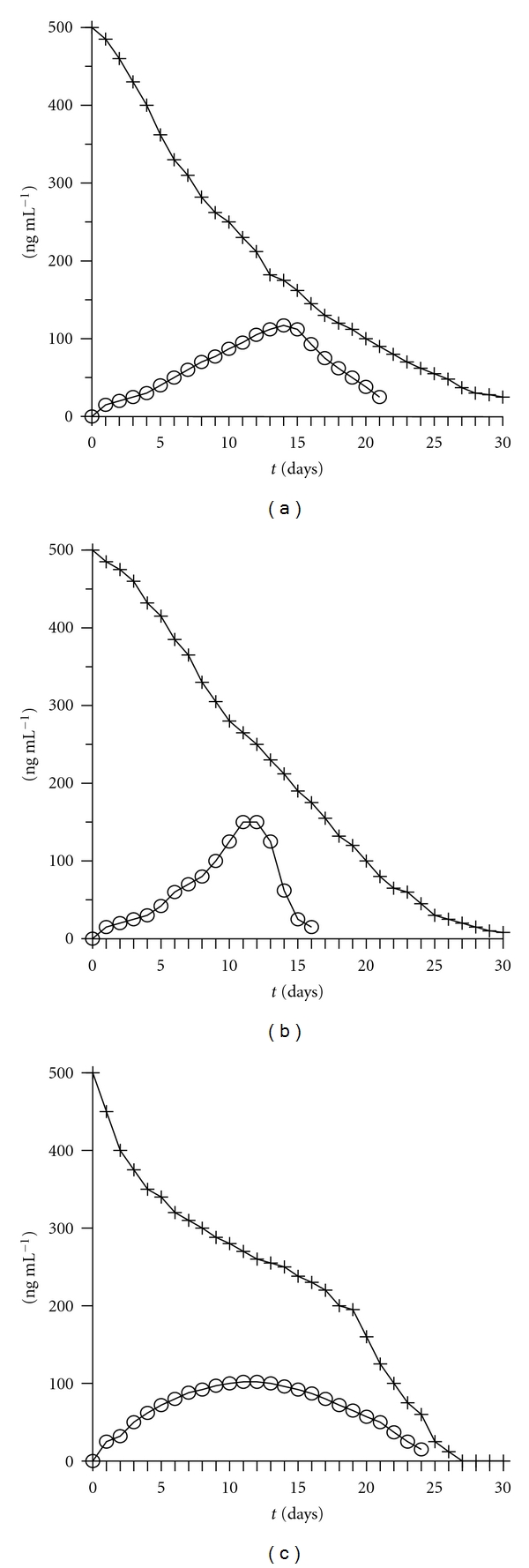
Curves showing the decomposition of carbaryl (+) and the formation of 1-naphthol (o), in (a) water, (b) soil, and (c) vegetables.

The micellar liquid chromatography method is also useful in photodecomposition studies of carbaryl in water to form 1-naphthol. Carbaryl, at a concentration of 500 ng mL^−1^, was added into natural water, and pH was adjusted to 7 (to force quick decomposition) and kept in closed transparent recipients. On a daily basis, 1 mL of water was removed and analysed to study the decomposition pattern.  Figure 2(a) shows the results obtained between days 0 and 30. In our laboratory, photodecomposition of carbaryl in water shows a half-life of 10 days.  Figure 1(a) illustrates the chromatogram obtained in the determination of carbaryl and 1-naphthol in water on day 10.

### 3.6. Decomposition of Carbaryl in Soil and Vegetables

Carbaryl was added in soil and vegetables in a ratio of 500 ng g^−1^ and mixed and kept in a transparent nonreactive container. Each day, a portion containing 1 g of soil or vegetables was taken, treated, and analysed. Figures 2(b) and 2(c) show the decomposition of carbaryl in soil and vegetables with a half-life of 12 and 15 days, respectively. The chromatogram obtained in soil on day 2 is shown in Figure 1(b).

## 4. Conclusions

The micellar liquid chromatography procedure developed herein is rapid and allows the simultaneous determination of carbaryl and 1-naphthol in water, soil, and vegetables matrices with high sensitivity. The designed method easily achieves a high sample throughput with a short preparation time, and proves useful to determine the content of both pesticides. The proposed chromatographic procedure provides good results to determine the pesticides in the matrices studied in terms of linearity, accuracy, precision, recoveries, robustness, and sensitivity at the ng mL^−1^ level. Compared to other methods developed for the determination of pesticides, micellar mobile phases are less flammable, less expensive, less toxic, and biodegradable and can cosolubilize hydrophobic and hydrophilic analytes in this kind of matrixes. The elution of hydrophobic and hydrophilic analytes in the same MLC run is possible without a gradient elution. The use of an interpretative optimization strategy in MLC also makes more efficient and reliable mobile phase selection.

## Figures and Tables

**Table 1:** Chromatographic parameters of the pesticides: retention factors (*k*), efficiencies (*N*), and asymmetry factors (*B/A*) for carbaryl and 1-naphthol in selected micellar mobile phases at pH 3 containing different surfactants (SDS, NCPC, or Brij-35) in the absence or presence of the organic solvent pentanol.

Mobile phase	Carbaryl			1-naphtol		
	*k*	*N*	*B/A*	*k*	*N*	*B/A*
SDS 0.05 M-pH 3	16	2500	1.5	41	4000	1.5
SDS 0.10 M-pH 3	10	2200	1.5	25	3500	1.5
SDS 0.15 M-pH 3	6	2000	1.4	18	3500	1.5

NCPC 0.02 M-pH 3	52	300	2	70	250	2.5
NCPC 0.10 M-pH 3	41	200	2.2	58	150	3.0

Brij-35 0.05 M-pH 3	45	1200	2	63	1000	2.0
Brij-35 0.10 M-pH 3	16	4000	1.3	23	3700	1.3

SDS 0.05 M-2% pentanol (*v/v*)-pH 3	9	3600	1.7	21	4800	1.6
SDS 0.05 M-6% pentanol (*v/v*)-pH 3	8	4300	1.2	14	3200	1.4
SDS 0.15 M-2% pentanol (*v/v*)-pH 3	8	2800	1.5	14	3600	1.5
SDS 0.15 M-6% pentanol (*v/v*)-pH 3	4	4500	1.2	7.7	4500	1.2

## References

[1] Environmental Protection Agency (1991). *U.S. EPA. EPA's pesticide programs*.

[2] Soriano JM, Jiménez B, Font G, Moltó JC (2001). Analysis of carbamate pesticides and their metabolites in water by solid phase extraction and liquid chromatography: a review. *Critical Reviews in Analytical Chemistry*.

[3] Atrache LLE, Sabbah S, Morizur JP (2005). Identification of phenyl-N-methylcarbamates and their transformation products in Tunisian surface water by solid-phase extraction liquid chromatography-tandem mass spectrometry. *Talanta*.

[4] Martínez Galera M, Parrilla Vázquez P, Martínez Vidal JL, Martínez Fernández J, Pascual Gómez JL (2004). Large-volume direct injection for determining naphthalene derivative pesticides in water using a restricted-access medium column in RPLC-LC with fluorescence detection. *Chromatographia*.

[5] Yu K, Krol J, Balogh M, Monks I (2003). A fully automated LC/MS method development and quantification protocol targeting 52 carbamates, thiocarbamates, and phenylureas. *Analytical Chemistry*.

[6] Özhan G, Topuz S, Alpertunga B (2003). A simple method for the determination of carbaryl and 1-naphthol in fruit juices by high-performance liquid chromatography-diode-array detection. *Journal of Food Protection*.

[7] Nunes GS, Marco MP, Ribeiro ML, Barceló D (1998). Validation of an immunoassay method for the determination of traces of carbaryl in vegetable and fruit extracts by liquid chromatography with photodiode array and mass spectrometric detection. *Journal of Chromatography A*.

[8] Massey KA, Van Engelen DL, Warner IM (1995). Determination of carbaryl as its primary metabolite, 1-naphthol, by reversed-phase high-performance liquid chromatography with fluorometric detection. *Talanta*.

[9] Lehotay SJ, Lightfield AR, Harman-Fetcho JA, Donoghue DJ (2001). Analysis of pesticide residues in eggs by direct sample introduction/gas chromatography/tandem mass spectrometry. *Journal of Agricultural and Food Chemistry*.

[10] Santos Delgado MJ, Rubio Barroso S, Toledano Fernández-Tostado G, Polo-Díez LM (2001). Stability studies of carbamate pesticides and analysis by gas chromatography with flame ionization and nitrogen-phosphorus detection. *Journal of Chromatography A*.

[11] Segura Carretero A, Cruces-Blanco C, Cortacero Ramírez S, Carrasco Pancorbo A, Fernández Gutiérrez A (2004). Application of micellar electrokinetic capillary chromatography to the analysis of uncharged pesticides of environmental impact. *Journal of Agricultural and Food Chemistry*.

[12] Wu YS, Lee HK, Li SFY (1998). Separation and determination of pesticides by capillary electrophoresis. I. Rapid separation of fifteen n-methylcarbamate insecticides via micellar electrokinetic chromatography. *Journal of Microcolumn Separations*.

[13] Bedair M, El Rassi Z (2002). Capillary electrochromatography with monolithic stationary phases: 1. Preparation of sulfonated stearyl acrylate monoliths and their electrochromatographic characterization with neutral and charged solutes. *Electrophoresis*.

[14] Kumar KS, Suvardhan K, Chiranjeevi P (2005). Preparation of reagents for the sensitive spectrophotometric determination of carbaryl in environmental samples. *Analytical Letters*.

[15] Portela DC, Pereira IMF, Paíga P, Delerue-Matos C, Vaz MCVF (2003). Amperometric and spectrophotometric determination of carbaryl in natural waters and commercial formulations. *Analytical and Bioanalytical Chemistry*.

[16] Daghbouche Y, Garrigues S, De La Guardia M (1995). Solid phase preconcentration-Fourier transform infrared spectrometric determination of carbaryl and 1-naphthol. *Analytica Chimica Acta*.

[17] Brena BM, Arellano L, Rufo C (2005). ELISA as an affordable methodology for monitoring groundwater contamination by pesticides in low-income countries. *Environmental Science and Technology*.

[18] Marco MP, Chiron S, Gascón J, Hammock BD, Barceló D (1995). Validation of two immunoassay methods for environmental monitoring of carbaryl and 1-naphthol in ground water samples. *Analytica Chimica Acta*.

[19] Esteve-Romero J, Carda-Broch S, Gil-Agustí M, Capella-Peiró ME, Bose D (2005). Micellar liquid chromatography for the determination of drug materials in pharmaceutical preparations and biological samples. *Trends in Analytical Chemistry*.

[20] Gil-Agustí M, Alvarez-Rodríguez L, Monferrer-Pons L, Bose D, Durgbanshi A, Esteve-Romero J (2002). Chromatographic determination of carbaryl and other carbamates in formulations and water using Brij-35. *Analytical Letters*.

[21] Berthod A, García-Álvarez-Coque MC (2000). *Micellar Liquid Chromatography*.

[22] Torres-Lapasió JR (2000). *Michrom Software*.

[23] ICH Harmonised Tripartite Guideline Validation of Analytical Procedures: Text and Methodology Q2 (R1). http://www.ich.org/products/guidelines/quality/quality-single/article/validation-of-analytical-procedures-text-and-methodology.html.

